# Communications, Immunization, and Polio Vaccines: Lessons From a Global Perspective on Generating Political Will, Informing Decision-Making and Planning, and Engaging Local Support

**DOI:** 10.1093/infdis/jix059

**Published:** 2017-07-01

**Authors:** Lisa Menning, Gaurav Garg, Deepa Pokharel, Elizabeth Thrush, Margaret Farrell, Frederic Kunjbe Kodio, Chantal Laroche Veira, Sarah Wanyoike, Suleman Malik, Manish Patel, Oliver Rosenbauer

**Affiliations:** 1 World Health Organization, and; 2 Gavi, the Vaccine Alliance, Geneva, Switzerland;; 3 Eastern and Southern Africa Regional Office, United Nations Children’s Fund (UNICEF), Nairobi, Kenya;; 4 Pan American Health Organization, Washington, D. C.;; 5 Programme Division, UNICEF, New York, New York;; 6 Task Force for Global Health, Atlanta, Georgia; and; 7 Western and Central Africa Regional Office, UNICEF, Dakar, Senegal

**Keywords:** Polio, eradication, poliovirus, communications, advocacy, immunization, vaccine, endgame, oral poliomyelitis vaccine, OPV, inactivated poliomyelitis vaccine, IPV.

## Abstract

The requirements under objective 2 of the Polio Eradication and Endgame Strategic Plan 2013–2018—to introduce at least 1 dose of inactivated poliomyelitis vaccine (IPV); withdraw oral poliomyelitis vaccine (OPV), starting with the type 2 component; and strengthen routine immunization programs—set an ambitious series of targets for countries. Effective implementation of IPV introduction and the switch from trivalent OPV (containing types 1, 2, and 3 poliovirus) to bivalent OPV (containing types 1 and 3 poliovirus) called for intense global communications and coordination on an unprecedented scale from 2014 to 2016, involving global public health technical agencies and donors, vaccine manufacturers, World Health Organization and United Nations Children’s Fund regional offices, and national governments. At the outset, the new program requirements were perceived as challenging to communicate, difficult to understand, unrealistic in terms of timelines, and potentially infeasible for logistical implementation. In this context, a number of core areas of work for communications were established: (1) generating awareness and political commitment via global communications and advocacy; (2) informing national decision-making, planning, and implementation; and (3) in-country program communications and capacity building, to ensure acceptance of IPV and continued uptake of OPV. Central to the communications function in driving progress for objective 2 was its ability to generate a meaningful policy dialogue about polio vaccines and routine immunization at multiple levels. This included efforts to facilitate stakeholder engagement and ownership, strengthen coordination at all levels, and ensure an iterative process of feedback and learning. This article provides an overview of the global efforts and challenges in successfully implementing the communications activities to support objective 2. Lessons from the achievements by countries and partners will likely be drawn upon when all OPVs are completely withdrawn after polio eradication, but also may offer a useful model for other global health initiatives.

In May 2012, the World Health Assembly (WHA) declared the completion of poliovirus eradication to be a “programmatic emergency for global public health” and called on the Director General of the World Health Organization (WHO) to develop a comprehensive polio endgame strategy [1]. The resulting Polio Eradication and Endgame Strategic Plan 2013–2018 [2] (hereafter, the “Endgame Plan”) was endorsed by WHO member states at the Sixty-sixth World Health Assembly in May 2013, who thereby approved the targets, goals, and timelines to secure a lasting polio-free world. The Endgame Plan seeks to simultaneously eradicate wild poliovirus (WPV) and eliminate the risk of vaccine-derived polioviruses (VDPVs). Of the 4 objectives of the Endgame Plan, the second requires countries to strengthen immunization programs, introduce at least 1 dose of inactivated poliomyelitis vaccine (IPV) into routine immunization, and withdraw oral poliomyelitis vaccine (OPV) from use, starting with the type 2 poliovirus component.

To oversee the timely implementation of all activities under objective 2 of the Endgame Plan, the Immunization Systems Management Group (IMG), cochaired by the WHO and UNICEF, was established in early 2013. The IMG formed a number of working groups covering a range of priority areas, one of which was communications. The Communications Working Group (CWG), also cochaired by the WHO and UNICEF, brought together a diverse set of skills in global and national communications and advocacy, representing public health partners and academic institutions (ie, the WHO, UNICEF; Emory University; the Centers for Disease Control and Prevention; Gavi, the Vaccine Alliance; the Task Force for Global Health; Rotary International; and the Bill and Melinda Gates Foundation). The CWG contributed to the success of objective 2 by driving all of the communications-related activities needed to generate awareness and political commitment, inform national decision-making and planning, and support in-country program communications and training efforts.

A number of timelines were also predetermined via the Endgame Plan. A deadline of December 2015 was set for the introduction of IPV in the 126 countries that were not already using this vaccine in routine immunization programs. As a result of significant efforts around the world, almost all countries have since added IPV into their routine immunization schedules within this time frame, although because of a global shortage of IPV, approximately 50 countries at low risk for polio transmission are experiencing delays in resupply or stockouts that are likely to persist until 2018. Pending certification of the eradication of type 2 poliovirus, a tentative timeline of April 2016 was scheduled (and confirmed in October 2015) for the globally synchronized switch from trivalent OPV (containing types 1, 2, and 3 poliovirus) to bivalent OPV (containing types 1 and 3 poliovirus) in all 155 OPV-using countries and territories.

The importance of communications to objective 2 of the Endgame Plan was evident from the start. To most individuals outside of the Global Polio Eradication Initiative, the new requirements for national immunization programs were an unexpected development. There was concern that these unforeseen activities would incur an additional commitment of human and financial resources within a short timeline, potentially displacing activities already planned. For both the introduction of IPV into routine immunization programs and the OPV switch, the scientific rationale was complex and could cause confusion or raise questions about the safety and effectiveness of OPV. The level of coordination and synchronization of activities required needed an unparalleled level of global consensus. The communications strategy therefore demanded quality planning and implementation, careful attention to the technical accuracy and clarity of messaging, and effective engagement with all key stakeholders, to achieve the understanding and commitment of all.

In its first months of operation, the activities of the CWG quickly focused on a number of core areas, as outlined in Figure 1. The first core area involved global-level communications and advocacy, to ensure that all partners and stakeholders within the spheres of polio eradication and immunization were knowledgeable and equipped to serve as active supporters. Second, for evidence-informed decision-making and planning, the CWG was responsible for developing and disseminating the documentation necessary to secure national commitment and drive operational planning. The third area involved national program communications and health worker training, to generate local awareness, engagement, and demand and acceptance for IPV. Finally, the CWG set up clear processes for priority setting, project management, accountability, and coordination.

**Figure 1.**
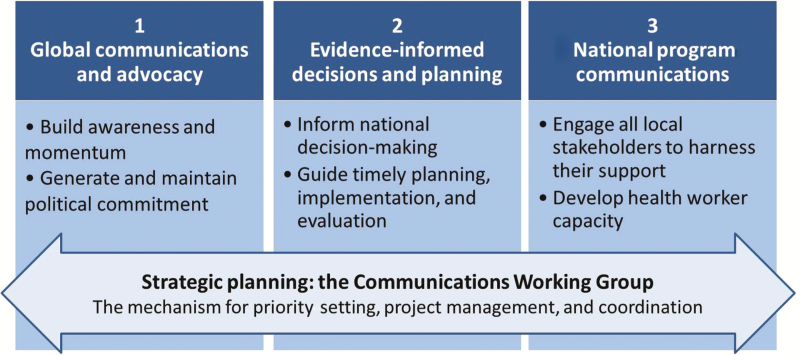
The core areas of focus of the Communications Working Group.

As the work of the CWG advanced over time, a standardized method was established for the planning, development, and dissemination of guidance and materials, together with regular forums for input and dialogue. This familiar mode of operation for all work (concerning IPV introduction and the OPV switch) offered the benefits of active participation of all key stakeholders and helped to build wide ownership for and uptake of the group’s outputs. Herein, we report on the strategic planning processes, technical approaches, and lessons learned by the CWG, which may be relevant to other global health initiatives with similarly complex mandates and accelerated timelines.

## GLOBAL COMMUNICATIONS AND ADVOCACY

At the beginning of preparations for both IPV introduction and the OPV switch, a detailed communications plan was generated by the CWG and reviewed by the IMG, outlining the core objectives, risks and opportunities, audiences, messaging, materials, timelines, and responsibilities. This planning process was particularly critical for harmonizing activities with the milestones of technical implementation and for anticipating and overcoming any possible challenges. It also ensured that all key stakeholders were actively engaged in a manner corresponding to their role and function, gaining their buy-in via early involvement in the planning process.

Working systematically, the first priority for the CWG was to build awareness of the Endgame Plan, initially at global and regional levels, to begin to activate the necessary support to countries. Accordingly, a layered series of communications materials were designed, in coordination with other IMG subgroups. The preliminary series was written in the simplest language and focused on establishing an overall understanding of the key steps, timelines, and basic rationale. This was followed by the creation of materials of increasing complexity that were targeted to specific audiences involved in decision-making, planning, or implementation. Throughout, references to the WHO governance and policy-setting mechanisms helped to reinforce commitment to the Endgame Plan. This included the WHA declarations and resolutions, the recommendations of the Strategic Advisory Group of Experts on Immunization (SAGE), and the WHO position papers on polio vaccines (updated in February 2014 and then again in March 2016 [3]). As a home for all communication materials and guidance, a web site [4] dedicated to objective 2 was launched at the outset and became a widely used resource.

In addition to the written communications materials, regular 2-way dialogue was an essential factor for achieving the targets of objective 2, in the form of workshops, trainings, regular teleconferences, and webinars. This approach not only helped to foster an understanding of the rationale and programmatic requirements, but also served a number of important purposes. First, the dialogue helped gather inputs and insights to inform later iterations of communications materials and programmatic guidance and to demonstrate via a feedback loop that global partners had genuinely listened. Second, the open discussions encouraged collaboration and cultivated trust among all partners and stakeholders (eg, by providing a platform for participants to voice concerns). Last, this approach offered a sense of shared responsibility in working toward the same goal, despite the Endgame Plan being a global initiative.

Specifically concerning the OPV switch [5] in 155 countries and territories in a 2-week period in April 2106, the unprecedented nature of this initiative represented a significant challenge for communications and advocacy efforts. The CWG started early in 2015 and proceeded methodically, developing a comprehensive plan and then a package of initial materials, including a high-level briefing note, an introductory PowerPoint presentation [6] to introduce the technical rationale and early considerations for national planning, and a frequently asked questions document. Given the potential risk of misrepresenting the global withdrawal of trivalent OPV, all messaging was carefully crafted and recognized as crucial to forming a common global vocabulary and understanding.

While global media engagement on the switch was generally very limited, a press release and media briefing was eventually conducted in mid-April 2016, accompanied by donor and partner briefings. These activities were primarily intended to offer recognition to all of the immunization personnel involved worldwide in the switch. The coverage that resulted surpassed all expectations, represented accurate messaging, and underscored the switch as a unique and global success story.

**Figure 2.**
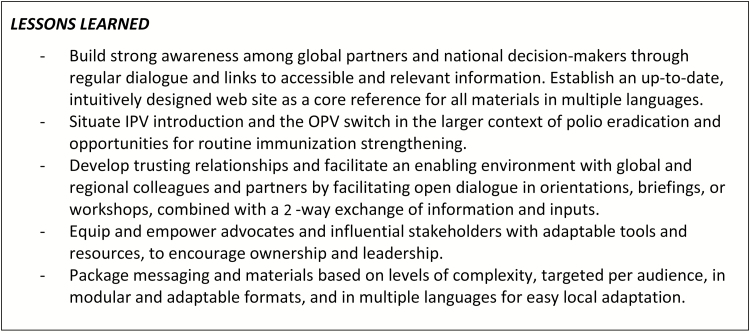
Lessons learned: global communications and advocacy. Abbreviations: OPV, oral poliomyelitis vaccine; IPV, inactivated poliomyelitis vaccine.

Several lessons that were learned from the implementation of the global communications and advocacy strategy for objective 2 are summarized in Figure 2.

## EVIDENCE-INFORMED DECISIONS AND PLANNING

A decision to introduce a vaccine into a national immunization program or to switch from one vaccine for another presents many issues for a country in prioritizing investments in the health sector. Traditionally, the key factors that are examined in making a decision about adding a vaccine include the public health priority of the target disease, economic and financial feasibility, target cohort and vaccination schedule, and impact on the immunization program and overall health system [7]. Evidence of disease burden is generally another consideration, but this was not the case for IPV. Rather than the typical use of vaccine to control or eliminate disease, the WHO recommended at least 1 dose of IPV as a strategy to mitigate the potential risk of reemergence of type 2 polio following the OPV switch [8].

For this reason, much of the overall decision to introduce IPV had been taken on behalf of countries through the global direction established in the Endgame Plan. Nevertheless, questions remained on specific timelines, schedules, and, in some cases, financing and procurement of vaccines, to be resolved through the relevant national mechanisms. Recognizing the importance of a systematic and transparent decision-making process to eventual community acceptance of the new vaccine, discussions on these outstanding topics helped to engage local leaders and stakeholders. To assist, a comprehensive information kit targeted to National Immunization Technical Advisory Groups, or their equivalent, was developed and disseminated, encompassing the WHO policy recommendations, evidence base, operational guidance, and supporting materials. Case studies and short documentary-style videos on the introduction of IPV in early adopter low- and middle-income countries were also seen to be particularly useful for sharing learning and best practices.

One approach that proved to be particularly effective in generating momentum for IPV introduction was the organization of region-specific workshops and consultant trainings in late 2014, followed by full-day sessions during regional immunization manager meetings in early 2015. These forums brought together multiple stakeholders, including program managers, senior staff from ministries of health and regional partner agencies (eg, from the WHO, UNICEF, and the Centers for Disease Control and Prevention), and national and regional technical experts, and resulted in the formation of networks for the sharing of evidence, guidance, and experiences. The discussions that took place were pivotal for offering a platform for voicing concerns and collective problem solving, cultivating an understanding of the rationale for IPV introduction and steps to implementation, exchanging relevant lessons learned from vaccine introductions to date, and informing the refinement and future directions of all communications materials and guidance produced by the CWG. Interestingly, these opportunities for dialogue often served to stimulate local innovations, in some cases shaped by global guiding principles, and further encouraged regional and national ownership and leadership.

For monitoring progress on national commitments, decisions to introduce IPV, and the eventual launch, a series of indicators were decided. Progress was tracked on a monthly basis and published on the objective 2 web site, offering transparency that may have reinforced commitments and generated a mutual accountability. Over time, issues emerged in areas such as demand for IPV and management of the eligible target population, multiple injections for countries administering at least 3 vaccines at the same visit, and reductions in IPV supply due to technical challenges in scaling up production. These presented both challenges and opportunities for the CWG and prompted the need to generate new guidance, training materials, health worker job aids, and other resources to assist countries. Case studies, which appear at the end of the body text, provide further details on specific thematic areas, such as managing demand for IPV, multiple injections, and the switch.

**Figure 3.**
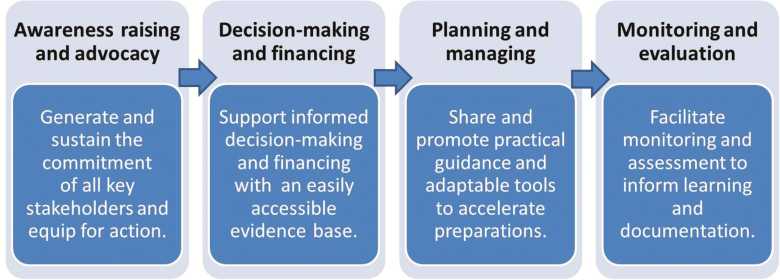
Phases of communications for the oral poliomyelitis vaccine switch.

**Figure 4.**
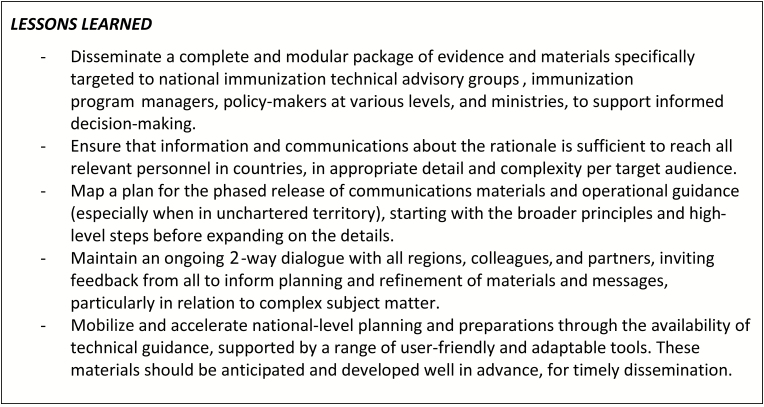
Lessons learned: evidence-informed decisions and planning.

While the initial phases of work were dedicated to raising awareness and informing decision-making, the accelerated timelines required that efforts rapidly move to focus on planning and preparations for implementing IPV and the OPV switch (Figure 3). The CWG was responsible for coordinating the development of guidance and various adaptable tools and templates [9] (eg, for budgeting, logistics, communications, and monitoring and validation), all designed to simplify and accelerate the national planning processes, and to offer sound programmatic recommendations as a basis for local implementation. Specifically in relation to the OPV switch, all materials were disseminated shortly after being tested during a series of dry runs (or switch pilots) in several countries in the second quarter of 2015. However, it was not until after October 2015, when SAGE definitively confirmed the switch window to last from 14 April to 1 May 2016, that in-country planning truly gained momentum. Given the switch’s unprecedented nature and degree of global synchronization, this was perhaps not unexpected. A summary of lessons learned for the communications activities related to packaging the evidence and informing decision-making and planning are summarized in Figure 4.

## NATIONAL PROGRAM COMMUNICATIONS

The program requirements of objective 2 of the Endgame Plan necessitated multiple levels of engagement with in-country stakeholders to achieve the awareness and acceptance that would be crucial to the success of IPV introduction and the OPV switch. Similar questions were being asked all around the world: why should our infants receive 2 vaccines, IPV and OPV, against the same disease? How are these vaccines different? Are 3 injections during a single visit risk free? Is OPV still safe and effective? Answers would eventually be locally adapted and context specific, ideally based on the findings of formative research or surveys. For example, at its simplest level, messaging on IPV described it as distinct but complementary to OPV and that IPV and OPV together provide the best protection against polio at this stage of global eradication.

To accelerate the implementation of local communications activities, a variety of globally developed guidance materials and adaptable templates on IPV [10] and the OPV switch [11] were widely disseminated in multiple languages and used by countries. This helped to maintain a consistent and accurate message, and it expedited national efforts to produce and finalize content. In-country activities also worked to ensure that local experts, medical associations, civil society, traditional leaders, and journalists would become partners and contributors to a positive communications environment.

Specific to the OPV switch, it is important to note the complexities of the rationale and potential for misinterpretation—unintentional or otherwise—of the vaccine safety–related reasons for withdrawal of trivalent OPV. At a global level, the general recommendation to countries was to avoid any proactive public communications but, instead, to focus on targeted engagement of well-informed and key stakeholders to contribute to an accurate local dialogue. Both content and format of the global communications materials (eg, technical briefs, high-level summaries, presentations, and frequently asked questions; long and short versions) and related planning guidance for countries were all developed in close consultation with technical experts and regional colleagues. The approach represented increasing layers of detail and offered adaptability for different audiences and local needs. It was later observed that, during implementation, most countries adopted the strategy to avoid any public communications, instead favoring more-nuanced and targeted approaches. For some regions, a convergence in timing with the annual immunization week in late April also required specific planning. Case study 3, below, provides a more detailed review of the messaging challenges related to the OPV switch.

Another vital activity was the training of health workers and other immunization personnel to build the skills and knowledge needed to correctly handle and administer the polio vaccines and capably explain the program changes to caregivers and local communities. Many studies [12],[13] have demonstrated that health professionals have a pivotal role in shaping parental attitudes toward immunization and maintaining trust in vaccines. For the complexities inherent to IPV introduction and the OPV switch, this included managing multiple injections, collecting and disposing of tOPV, and responding to questions from caregivers. Thus, for program managers, it would be a priority to implement quality and timely training and to ensure adequate monitoring and supervision.

Cross-cutting all of the communications, stakeholder engagement, and capacity-building activities were the opportunities to strengthen routine immunization programs. Despite the extremely compressed timelines and potential risks of diluting or distracting from the core objectives of IPV introduction and the OPV switch, it is possible that gains have been made, even if only as an indirect outcome. Anecdotal reports so far indicate that the intensity of technical assistance, specialist guidance, and targeted catalytic financing that have been made available since 2014 for objective 2 have contributed to reinforcing country-level knowledge and practices related to communications. Studies are planned for 2017 to further investigate and measure the wider impact of these efforts.

**Figure 5.**
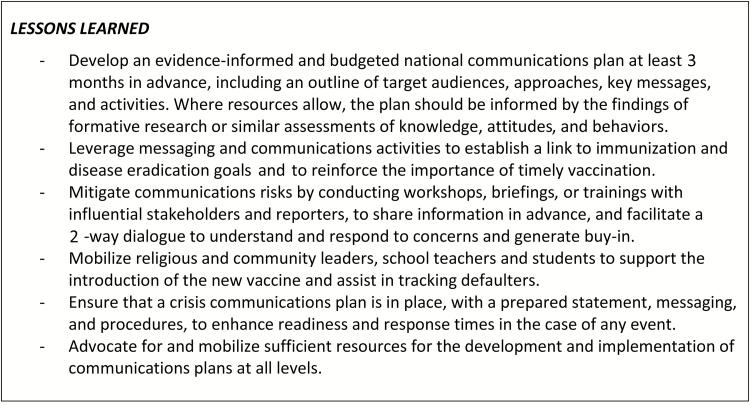
Lessons learned: national program communications.

Lessons that were learned through the support to in-country communications and training are summarized in Figure 5.

## STRATEGIC PLANNING: THE CWG

The contributions and commitment of all CWG members were critical to its achievements and learning. Foremost of note is the diverse capabilities that existed within the CWG. This group of individuals represented a wide range of organizations, geographies, and types of specialist communications experience, further enhanced by the practical and enthusiastic nature of many. During its years of operation, the evolving relationship between organizations contributed to neutralizing any organization-first mentality. On the other hand, continuity was at times a challenge and was fortunately mitigated by the flexibility and adaptability of many members. Overall, these factors contributed to forming a strong foundation of expertise that was able to adapt to evolving needs and manage a wide range of topics.

**Figure 6.**
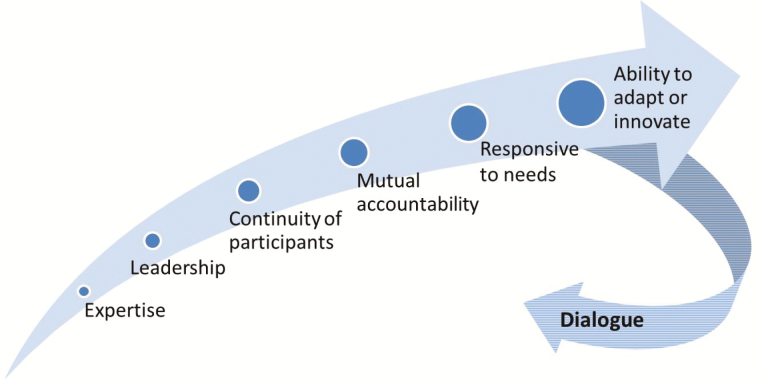
Factors that contributed to the success of the Communications Working Group.

In terms of project management, the CWG was facilitated by fortnightly teleconferences to share updates, routinely review the latest work plan and status of activities, explore technical questions or insights from regions or countries, and rapidly resolve any issues. The format of the work plan, at times covering >50 different activities, was established as a collective tool and brought transparency and accountability to the responsibilities and timelines that were listed. In late 2013 and mid-2014, 2 in-person meetings were held to foster strong interpersonal relations, and many members participated in the biannual IMG in-person meetings. The emphasis on participatory dialogue, also applied to the operations of the CWG, helped to ensure its effectiveness in engaging all partners, seeking and acting on inputs, and in producing quality outputs (Figure 6).

While an evaluation of the work of the CWG and its outputs is planned for 2017, the emphasis on 2-way dialogue and feedback loops integrated into the processes of document development and dissemination stimulated the timely inputs necessary to inform final refinements. For now, the measures of its effectiveness may be assessed by the commitment of effectively all countries to introduce IPV and implement the OPV switch. Analyses of global media coverage, representing >70 articles on the OPV switch, showed that global media featured accurate and accessible explanations of the switch, in part because of the longstanding relationships with many key health journalists. Additionally, reporting from regional and country-based partners affirmed the wide uptake and use of all CWG materials and templates.

**Figure 7.**
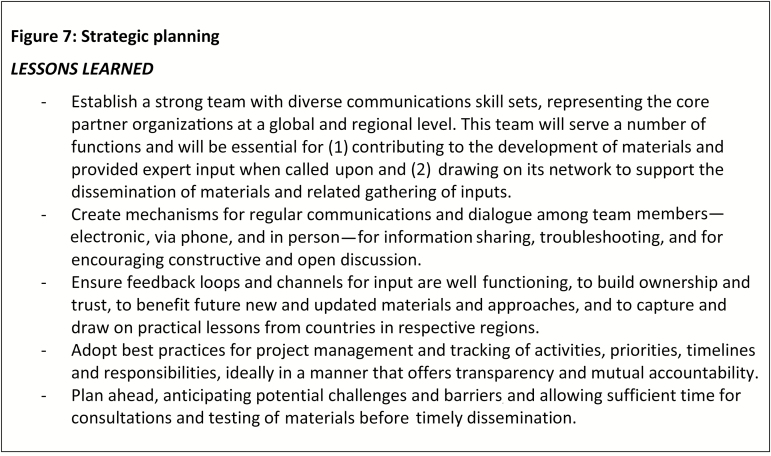
Strategic planning.

The combination of strategic planning, open management processes, and robust expertise combined to ensure that the group was able to drive forward on a significant program of work. The knowledgeable leadership and coordination of the IMG, to which the CWG reported, further stimulated a true collaboration and influenced close coordination between the CWG and other IMG subgroups. Overall, it was a unique and fruitful blend of characteristics that contributed to the success of this group. The lessons learned in relation to strategic coordination of the CWG at a global level are outlined in Figure 7.

## CONCLUSIONS

The introduction of IPV and the OPV switch through the Endgame Plan built on communications lessons from previous efforts to introduce new vaccines to routine immunization programs and may contribute new lessons with the potential to guide future similar global initiatives. The compressed timelines, ambitious targets, and inherent complexities of these activities made it a challenge to convince countries and achieve the global consensus necessary that the plan should be implemented within the set window. While the success of the milestones achieved proves that such a globally coordinated initiative is feasible, the lessons raise a number of important considerations for the role of global-level communications in building awareness and commitment, informing national decision-making and planning, and building local-level support and acceptance.

Central to the success of the communications function in driving this program was its ability to generate a meaningful policy dialogue about polio vaccines and routine immunization. This dialogue was vital to fostering stakeholder participation and ownership, strengthening partner coordination, guiding implementation, and facilitating an iterative process of feedback and learning. Informing these interactions was a comprehensive package of materials and messaging that traversed a diverse set of areas (eg, program planning, financing, communications, training, logistics, and monitoring), in an array of adaptable formats, and covered the spectrum from simple to scientific. In addition to reaching its programmatic goals, the ripple effect on reinforcing technical capacity at all levels should be noted, even if only an indirect outcome.

Beyond 2016, the level of intensity of the CWG will decrease, but its continued functioning will remain crucial to ongoing issues, particularly those related to the global IPV supply constraints. A new communications plan has been developed to help manage information and expectations in the post-tOPV era, during which will exist the risk of vaccine-derived poliovirus outbreaks, continued IPV shortage, and remaining needs to document and promote the lessons learned. This latest phase of work will ensure that communications plans and messaging are in place to respond effectively to any events that may impact objective 2 or have wider implications for confidence in the Endgame Plan and immunization more generally. These efforts will contribute to securing the gains made by countries in stopping polio transmission and preparing for full OPV cessation, as the world moves into the final stages of eradication.

## CASE STUDIES

The following case studies are intended to provide a more in-depth review of specific topic areas where tailored communications strategies were a key factor to their success.

### Case Study 1. Managing Demand for IPV and OPV: 2 Vaccines Against Polio

The need to create the right level of demand for IPV (1 dose administered at 14 weeks of age or the nearest visit) posed specific communications challenges. Unlike the introduction of other new vaccines, such as pneumococcal or rotavirus, IPV was not being added to respond to a specific disease burden. The rationale for IPV was unique in serving as a risk mitigation strategy associated with the eventual withdrawal of all OPVs and the possible reintroduction of polioviruses after the OPV switch. Further, a range of questions were expected from caregivers and communities, such as why a second vaccine against polio is being administered, why and how IPV is different to OPV, and why IPV is being introduced in the absence of disease burden. 

To assist countries in tackling these challenges, a range of templates and materials were developed at a global level to help accelerate in-country efforts and to support the accuracy and consistency of messaging. These resources and recommendations were produced through the CWG in close collaboration with global technical experts. They emphasized matching the right level of information to specific target audiences and precisely wording content on rationale and risks, such as ensuring that IPV was seen as an added benefit. 

An added focus was placed on capacity building for health workers, given that they may represent the most trusted source of information on immunization. Related training materials emphasized the importance of administering IPV at the right age, clearly and simply explaining the rationale for IPV, and strengthening interpersonal communications skills, particularly in listening and reassuring caregivers and communities. 

Accordingly, in many countries, launch events and related public communications to mark the introduction of IPV were intentionally minimized, to avoid overpromotion of the vaccine and any potential consequences for demand among ineligible birth cohorts. National communication plans for IPV were therefore locally adapted and designed to balance these various objectives. Together, these approaches enabled countries to find the right strategy for generating the appropriate level of demand for IPV.

### Case Study 2. Multiple Injections in a Single Visit

Many countries administer multiple vaccine injections (including >3 injections) to infants in a single visit and achieve high vaccine coverage and acceptability. Other countries, particularly low-income and middle-income countries, are in the process of introducing additional vaccines into their routine immunization schedule, including pneumococcal and IPV, making 3 injections during a single visit an increasingly common occurrence. In this context, in planning to add IPV to routine schedules, some countries raised concerns about multiple vaccine injections in a single visit. 

In 2015, these concerns led to a systematic review of evidence [14] on the safety of administering multiple injectable vaccines during a single visit (specifically for IPV and pneumococcal and pentavalent vaccines). The review showed that, when given together, these vaccines are safe and generally well tolerated by infants. Data support coadministration of these vaccines, and no increase in reactogenicity was found when compared with vaccines injected in separate visits. 

Studies [15] indicated that parental acceptance of all injections was associated with a positive recommendation by the health worker and an expression of concern about the severity of disease against which the child was being vaccinated. Furthermore, study findings demonstrated providers often overestimated the concerns of parents and caregivers about multiple injections, indicating that these concerns can be addressed with effective communication and immunization practices, with implications for health worker training [16].

The conclusions and recommendations from the SAGE meeting in April 2015 [17] that assessed the outcomes of the systematic review, and an article published in 2014 on provider and parental attitudes and practices [18], were both key to informing the development of materials by the CWG to assist countries in managing the acceptance of multiple injections with IPV. Materials targeted policy-makers and program managers to inform their decisions on the timing of IPV administration, the need to implement any rapid preintroduction research on health worker practices and caregiver attitudes, and the various training and communication resources for health workers. Given the central role of health workers in providing the necessary reassurances on safety and correct vaccine administration, using appropriate pain-reduction techniques [19], a specific training module and job aid [20] were also included in the overall global package. 

### Case Study 3. Messaging for the OPV Switch: Maintaining Confidence in OPV

OPV is one of the safest and most successful vaccines available, and its large-scale administration has resulted in the reduction of polio disease by >99% over the past 30 years. tOPV has been the formulation traditionally used. Its rare association with circulating VDPVs led to the public health policy decision to remove and replace it with bOPV in a globally synchronized switch in April 2016.

Careful messaging was developed on why tOPV was being replaced by bOPV, to communicate the rationale to global and regional stakeholders, national policy-makers, healthcare experts, and community-level partners and health workers. Wording was selected to achieve an ease of comprehension for the intended target audience. The risk of circulating VDPVs was put into a public health context (ie, by underscoring that the benefits of tOPV were now clearly outweighed by the small risk of circulating VDPVs). For the most basic materials [21], the switch was simply framed as a transition in vaccines used at this stage of polio eradication, before withdrawal of all OPVs.

Proactive public communications at a community level were minimized, with tactics instead focusing on advance engagement of local stakeholders (eg, experts, medical associations, civil society, and journalists) and preparedness for reactive messaging. These activities helped to establish and maintain a favorable environment and were situated in the context of polio eradication and the importance of immunization in general.

Following the switch, no communications issues were reported, and an analysis of all national and global media coverage determined that all messaging was accurate. Vaccine acceptance rates remain high, with no decline associated with the change from tOPV to bOPV.

## Notes

***Disclaimer.*** The findings, interpretations and conclusions expressed in this report are those of the authors and do not necessarily reflect the policies or views of the World Health Organization, the United Nations Children’s Fund, the Bill and Melinda Gates Foundation, or the Centers for Disease Control and Prevention.

***Financial support.*** This work was supported by the Bill and Melinda Gates Foundation (grant OPP1095024 to M. M. P., C. L. V., and S. W.).

***Supplement sponsorship***. This work is part of a supplement coordinated by the Task Force for Global Health with funding provided by The Bill and Melinda Gates Foundation and the Centers for Disease Control and Prevention.

***Potential conflicts of interest.*** All authors: No reported conflicts. The author has submitted the ICMJE Form for Disclosure of Potential Conflicts of Interest. Conflicts that the editors consider relevant to the content of the manuscript have been disclosed.
